# Green transformational leadership and employees’ green behavior: a cross-level moderated mediation study

**DOI:** 10.3389/fpsyg.2026.1720404

**Published:** 2026-02-10

**Authors:** Jianwei Cao, Haoxiang Sun, Xulei Cao, Haoran Ma

**Affiliations:** School of Business, North Minzu University, Yinchuan, China

**Keywords:** cross-level, employees’ environmental passion, employees’ green behavior, green transformational leadership, pro-environmental organizational climate

## Abstract

Employees’ green behavior directly contributes to corporate environmental sustainability and generates positive spillover effects on society that support broader green transformation. As a leadership style that encourages employees’ pro-environmental actions, green transformational leadership has attracted growing scholarly attention. However, the mechanisms through which green transformational leadership shapes employees’ green behavior remain underexplored. Drawing on social learning theory and affective events theory, this study developed a cross-level theoretical model linking green transformational leadership and employees’ green behavior. Using Mplus, multilevel analyzes on matched data from 325 employees nested within 53 teams were conducted. The results indicated that (1) green transformational leadership was positively related to employees’ green behavior; (2) employees’ environmental passion mediated the relationship between green transformational leadership and employees’ green behavior; (3) pro-environmental organizational climate negatively moderated the mediating effect of employees’ environmental passion on the relationship between green transformational leadership and employees’ green behavior, such that the indirect effect is weaker when the organizational climate is more strongly pro-environmental. These findings enhance understanding of the mechanisms through which green transformational leadership shapes employees’ green behavior and offer practical implications for leveraging leadership to promote pro-environmental actions in organizations.

## 1 Introduction

As awareness of the severity of environmental problems has intensified, a growing global consensus has emerged regarding the urgent need to develop and implement a sustainable development paradigm. A green development model not only responds to the global imperative of environmental protection, but also safeguards the long-term sustainability of economic activity. Therefore, this model has attracted increasing attention from countries and regions worldwide. Within contemporary market economies, firms acting as key actors bear a social responsibility to advance green development. Integrating green development strategies into firms’ core business processes has become a critical means of fulfilling environmental responsibilities and facilitating society-wide transitions toward environmentally friendly development. The implementation of firms’ green strategies ultimately depends on employees’ green behavior (hereafter, EGB) at the micro level to reduce adverse environmental impacts directly determine the effectiveness of corporate environmental management (Stern, 2000). Consequently, employees’ green behavior plays a central role in operationalizing organizational green practices and generates positive spillover effects that contribute to broader social transformation (Wu et al., 2022). Accordingly, elucidating the mechanisms that give rise to EGB is essential for establishing a theoretical foundation to guide managerial practices aimed at promoting such behaviors.

Existing research has identified antecedents of employees’ green behavior at both the individual and organizational levels. At the individual level, scholars have primarily examined how factors such as work engagement (Wu et al., 2022), personal values, and a sense of responsibility (Wu et al., 2019) shape employees’ green behavior. At the organizational level, studies have largely focused on the promotive effects of green human resource management (Rubel et al., 2021; Yadate, 2024), green transformational leadership (Lathabhavan and Kaur, 2023), and organizational green culture (Azhar and Yang, 2022) on EGB. Notably, green transformational leadership (hereafter, GTL) is a leadership style that mobilizes and motivates employees to engage in environmentally responsible actions (Robertson, 2018), and it plays a critical role in managerial practices aimed at advancing corporate environmental performance. Leaders play a central role in the formulation, implementation, and supervision of corporate green strategies. Accordingly, whether employees engage in green behaviors depends substantially on whether leaders endorse and practice green management principles (Farrukh et al., 2022). GTL embeds environmental protection into the core of decision-making, and through behavioral modeling and value articulation, fosters employees’ identification with and commitment to environmental initiatives. Compared with conventional transformational leadership, GTL exhibits a stronger environmental orientation in goal-setting, incentive mechanisms, and value transmission, thereby more effectively promoting the emergence and persistence of EGB.

A closer synthesis of the literatures indicates that prior investigations into the effects of GTL on EGB have identified several internal mechanisms and boundary conditions, yet notable gaps persist. First, with respect to mediating mechanisms, extant studies have examined the issue from perspectives such as intrinsic and extrinsic motivation, resource integration, and green human resource management, and have identified mediators such as creative process engagement (Mittal and Dhar, 2016), green organizational identification (Al-Ghazali et al., 2022), values (Wang et al., 2018), and environmental concern (Robertson and Barling, 2017) in the relationship between GTL and EGB. However, comparatively little attention has been devoted to the affective, leaving the emotional pathways through which GTL exerts influence EGB underexplored. Second, regarding boundary conditions, the literatures to date has predominantly examined individual-level moderators, such as value congruence (Wang et al., 2018), green identity (Du and Yan, 2022), environmental attitude (Khan and Khan, 2022), green self-efficacy (Chen and Wu, 2022), environmental concern (Liu and Yu, 2023), humane orientation (Huang et al., 2023), and work engagement (Zaid and Yaqub, 2024), that shape the relationship between GTL and EGB and its underlying mediating processes. By contrast, fewer studies have systematically incorporated organization-level shared perceptions or situational strength, limiting insight into how organizational context may cross-levelly shape the translation from individual psychological states to observable pro-environmental actions. Accordingly, the field would adopt cross-level organizational–individual approaches to rigorously examine the affective mechanisms by integrating an affective perspective, one that explicates proximal emotional drivers of behavior, and delineate boundary effects across differing organizational contexts. Such an integrative approach is essential for providing a more comprehensive account of the causal chain through which GTL elicits EGB and for offering empirically grounded, organization-level guidance for targeted managerial interventions.

Social learning theory posits that individuals acquire behavioral patterns, value judgments, and skills not only through direct experience, but also through observing and imitating the actions of others (Bandura, 1977). Within organizational settings, transformational leaders—by virtue of their charisma, inspirational appeal, intellectual stimulation, and individualized consideration—often serve as salient referents for employees’ observation and learning (Bass, 1999). When GTL explicitly articulate environmental values, model environmentally sound practices, and publicly reward pro-environmental behaviors, employees are likely to adopt these behaviors through an “observe–evaluate–imitate” process, viewing leaders’ green actions as acceptable and implementable behavioral templates. Thus, social learning theory provides a strong theoretical foundation for explaining the relationship between GTL and EGB. Drawing on this perspective, the present study empirically examines the direct effect of GTL on EGB within the Chinese context.

Affective events theory posits that significant events in the work environment (such as leader behaviors) elicit affective reactions that subsequently influence employees’ attitudes and behaviors (Weiss and Cropanzano, 1996). In environmental contexts, employees’ environmental passion (hereafter, EEP) is defined as a high-arousal positive affect directed toward environmental goals (Chen et al., 2015; Vallerand et al., 2003), reflecting individuals’ emotional engagement and motivational readiness when confronted with green visions or exemplars (Ashkanasy and Humphrey, 2011). As an affective construct, EEP not only captures an immediate psychological response to situational stimuli, but also possesses the capacity to be translated into behavioral impetus (Vandercammen et al., 2014), as high-arousal positive affect facilitates the prioritization of action and enhances behavioral persistence. As an extension of transformational leadership within the environmental domain, GTL emphasizes that leaders stimulate EEP by demonstrating environmental awareness, advocating green ideals, and enacting ecological behaviors, thereby encouraging employees’ proactive participation in environmental practices. Accordingly, grounded in affective events theory, EEP can be conceptualized as the affective mechanism through which GTL is translated into employees’ concrete green behaviors. Accordingly, this study adopts EEP as the affective mediator to probe the internal mechanism linking GTL to EGB.

Affective events theory further emphasizes that features of the work environment determine how events are appraised and interpreted, which in turn influences the intensity of affective responses and the likelihood that affect will be translated into behavior (Weiss and Cropanzano, 1996). Whether EEP ultimately translates into EGB depends on organizational-level interpretations and the degree of contextual support. Organizational context is not an abstract backdrop but is instantiated through members’ shared understandings of values and norms. Such organizational-level shared perceptions at the organizational level govern how employees construe events and emotions and influence whether affect can be mobilized into action (Schneider et al., 2013). A pro-environmental organizational climate (hereafter, PEOC) represents a prototypical form of such shared perception, aligning members around common cognitions regarding environmental values, norms, and practices (Norton et al., 2014), thereby shaping the strength of the pathway from EEP to EGB within the GTL process. Consequently, within the affective events theory framework, examining the relationships among PEOC, GTL, EEP, and EGB facilitates a deeper understanding of how organizational context cross-levelly moderates the formation and translation of affect into behavior. Accordingly, in the present study, PEOC is specified as a cross-level moderator to elucidate the boundary conditions of the “GTL → EEP → EGB” pathway.

In sum, this study integrates social learning theory and affective events theory to construct and test the affective mechanisms and boundary conditions through which GTL influences EGB. Specifically, social learning theory explains the direct effect of leadership—via charisma, inspirational appeal, intellectual stimulation, and individualized consideration—on subordinates’ behavioral tendencies, thereby providing theoretical justification for the main effect of GTL. Affective events theory explicates how work events trigger immediate emotional responses that shape subsequent behavior and identifies contextual features as critical determinants of affective intensity, thereby supporting the examination of EEP as a mediator and PEOC as a moderator. Drawing on these complementary perspectives, the present study positions GTL as the antecedent and EGB as the outcome, with EEP serving as an affective mediator and PEOC operating as a cross-level moderator, thereby systematically testing the leader–affect–behavior pathway and its variability across organizational contexts.

This study employs matched multilevel data from 53 teams and 325 employees to empirically test the relationships among GTL, EGB, EEP, and PEOC. The results confirmed a positive effect of GTL on EGB, identified EEP as a mediating mechanism linking GTL to EGB, and demonstrated that PEOC negatively moderates the mediating effect of EEP. Compared with extant research, the present study offers several incremental yet meaningful theoretical contributions to the literature.

First, this study enriches empirical research on the role of green transformational leadership in shaping employees’ green behavior within the Chinese context. Prior studies on GTL have predominantly relied on samples from western developed countries (Abourokbah et al., 2024; Rietze et al., 2025; Robertson and Barling, 2013), while empirical evidence from developing countries remains relatively limited. Given that workplace culture and value systems may differ substantially across national contexts, the effectiveness and underlying mechanisms of green leadership may also vary accordingly. By drawing on a Chinese sample, the present findings not only provide contextualized evidence from China but also further support the applicability of Social Learning Theory in explaining how GTL influences EGB.

Second, grounded in Affective Events Theory, this study identifies EEP as a key mediating mechanism linking GTL to EGB. Existing research on the GTL–EGB relationship has primarily focused on cognitive and motivational pathways, such as value congruence (Wang et al., 2018), green dedication (Khan and Khan, 2022), environmental commitment (Liao, 2024), and green intrinsic motivation (Kaya and Atsan, 2025), while paying relatively limited attention to the dynamic role of affect in this leadership–behavior process. Compared with these constructs, EEP represents a goal-directed, high-arousal affective state that emphasizes employees’ emotionally energized engagement with environmental issues during the enactment of green behaviors. EEP does not merely capture whether employees endorse environmental values or experience satisfaction from engaging in green actions; rather, it reflects the activated emotional energy and sustained enthusiasm that continuously motivate employees to invest effort in environmental behaviors. Because of this proximal and dynamic nature, EEP can drive green behavior even in the absence of strong external incentives or formal institutional constraints. Although prior studies have examined EEP as an important motivational mediator in the context of environmentally specific servant leadership (Yuan and Li, 2023), such explanations cannot be directly extended to capture the more complex affective mechanisms through which green transformational leadership operates. By conceptualizing GTL as a salient affective event and EEP as employees’ proximal emotional response to that event, this study elucidates how leadership behaviors activate employees’ emotional energy and translate it into sustained green behavior. In doing so, the findings complement predominantly cognition- and motivation-oriented explanations and extend the application of Affective Events Theory to the domains of green behavior and sustainability.

Third, building further on Affective Events Theory, this study incorporates PEOC as a cross-level contextual moderator, thereby revealing how the effects of green transformational leadership on employees’ green behavior vary across organizational contexts. While prior research has generally viewed PEOC as a uniformly positive antecedent of EGB (Zafar et al., 2024; Katz et al., 2022; Norton et al., 2017), it has largely overlooked the possibility of diminishing marginal effects under certain conditions. The finding indicates that PEOC may exert a crowding-out effect on GTL (Frey and Jegen, 2010). In high-PEOC contexts, organizations continuously transmit strong situational cues—through institutionalized norms, stable value consensus, and clear behavioral expectations—that pro-environmental behavior constitutes a taken-for-granted organizational standard. As a result, the marginal effectiveness of affective activation through leadership behaviors may be weakened. This negative moderating pattern departs from the conventional assumption that supportive climates invariably strengthen leadership effects and instead highlights a diminishing marginal utility of leadership influence in strong pro-environmental climates. By doing so, this study offers a novel theoretical perspective on the interaction between organizational climate and leadership behavior within Affective Events Theory: when organizational-level environmental cues (such as PEOC) are sufficiently salient and explicit, employees may rely more on default contextual guidance, and the affective activation triggered by leadership behaviors (such as GTL) may be attenuated rather than amplified. Conversely, in low-PEOC contexts, stronger leadership advocacy becomes particularly critical for stimulating employees’ emotional engagement and translating it into green behavior.

The remainder of this paper is arranged as follows. First, develop hypotheses and present the theoretical model. Second, describe the data collection procedures and variable measurements. Third, report the data analysis and empirical results. Finally, discuss the findings, derive managerial implications, and outline the limitations and directions for future research.

## 2 Research hypotheses and theoretical model

### 2.1 Main effect of GTL on EGB

Based on social learning theory, individuals acquire behaviors not only through direct experience but also through observing, imitating, and evaluating the actions of salient role models (Bandura, 1977). Within this framework, GTL conveys clear environmental signals to subordinates through its four core dimensions—charisma, inspirational motivation, intellectual stimulation, and individualized consideration—and promotes employees’ green learning and behavioral change through demonstration, motivation, empowerment, and catalytic processes. Specifically, charisma establishes a powerful role-model effect by signaling value congruence and moral commitment (Shamir et al., 1993). Leaders’ pro-environmental actions become focal objects of employee observation and imitation; during this observational process, employees not only perceive concrete behavioral exemplars but also, through affective resonance, internalize these behaviors as personal standards (Kim et al., 2017). This internalization, deepens employees’ appreciation of the value of green practices, heightens environmental sensitivity, and motivates similar behaviors. Second, when leaders exhibit inspirational motivation, they leverage mechanisms of imitation and identification to steer employees toward prioritizing sustainable choices in decision making (Bass and Avolio, 1993). Inspirational communication and the articulation of a shared green vision render abstract environmental goals concrete and collectively meaningful. Through observation and interaction, employees develop a collective sense of mission, which strengthens intrinsic motivation and prompts proactive proposals for green improvements (Norton et al., 2017). Through this social learning process, EGB becomes embedded in organizational norms rather than remaining merely an individual choice. Third, leaders who provide intellectual stimulation encourage employees to question existing practices and propose innovative solutions (Shalley and Gilson, 2004). Intellectual stimulation prompts deeper reflection on the imitation process, leading employees to attend not only to surface behaviors but also to the environmental implications underpinning those behaviors. Encouraged by leaders’ support and feedback, employees explore new green procedures and accumulate practical experience in addressing environmental problems, thereby reinforcing identification with green practices. Fourth, leaders’ individualized consideration creates a safe learning environment in which employees feel comfortable voicing concerns and ideas, thereby deepening their understanding of EGB through social interaction (Robertson and Barling, 2017). Individualized care—manifested as attention to individual needs, timely feedback, and emotional recognition—enhances employees’ sense of responsibility and intrinsic motivation, enabling EGB to transcend extrinsic incentives and become spontaneous, internalized, and persistent. In sum, GTL, through the synergistic operation of these four dimensions and via a leader–employee modeling–imitation–internalization social learning process, facilitates the translation of observed leadership green practices into EGB.

Hypothesis 1 (H1): GTL positively affects EGB.

### 2.2 Mediating effect of EEP

To elucidate the affective pathway through which GTL promotes EGB, this study selects EEP as a mediating variable. According to affective events theory, concrete events in the workplace trigger immediate affective reactions through individuals’ appraisal of the events’ importance and goal relevance (Weiss and Cropanzano, 1996). These immediate affective reactions can directly influence employees’ attitudes and behavioral tendencies in the short term; when similar affective experiences recur or persist, they may accumulate and become integrated into relatively stable affective states or dispositions. Within the affective events theory framework, leaders’ behaviors are commonly perceived by employees as affectively significant work events that elicit immediate emotional responses via individual appraisal processes (Robertson and Barling, 2013). Within this affective chain, EEP is conceptualized as the result of accumulated and integrated positive affective responses triggered by leadership, constituting a key affective output. Specifically, in green contexts, GTL concretizes green visions, emphasizes their moral legitimacy and organizational value, and models pro-environmental behaviors, thereby strengthening affective cues related to environmental issues. Upon receiving these cues, employees are more likely to appraise the events as important and positive, thereby experiencing high-arousal positive affect such as excitement, pride, and a sense of mission (Fehr et al., 2014; Mittal and Dhar, 2016). When employees perceive that leader-generated are highly relevant to their personal goals and possess positive valence and collective significance, repeated high-arousal positive affect is more readily consolidated and gradually transformed into a relatively stable affective state oriented toward environmental goals—namely, EEP. Affective events theory further posits that situational events and cues (e.g., leaders’ communication and modeling) shape employees’ immediate affective states, which in turn influence subsequent attitudes and behaviors (Weiss and Cropanzano, 1996). Empirical studies have demonstrated that different affective states elicit distinct behavioral patterns among employees (Christensen et al., 2018; Griffin et al., 2010; Hill et al., 2020). EEP is defined as a high-arousal, positive, and relatively enduring affective and motivational investment directed toward environmental goals; its energy-mobilizing function can convert affective arousal into behavioral engagement (Chen et al., 2015). Employees who experience EEP gain increased behavioral drive and action intentions via this mobilizing function, thereby raising the likelihood that they will proactively implement energy-saving and emission-reduction practices, propose green initiatives, and engage in other forms of EGB in daily work. Moreover, sustained affective investment helps align attitudes and behaviors, making employees more inclined to choose options consistent with their EEP when making work-related decisions. Prior evidence indicates that when EEP is stably elicited by leader-generated green events, employees are more likely to use this high-arousal positive affect as kinetic energy to commit to environment-related tasks and situational choices, producing EGB that align with the organization’s long-term interests (Bissing-Olson et al., 2013). In sum, when leaders display a GTL style, they tend to enhance employees’ affective orientations toward organizational environmental issues and, through the elicited EEP, stimulate more proactive EGB.

Hypothesis 2 (H2): EEP mediates the relationship between GTL and EGB.

### 2.3 Moderated mediation effect of PEOC

To illuminate how the mediating effect of EEP on the relationship between GTL and EGB varies across organizational contexts, this study selects PEOC as the moderating variable. Grounded in affective events theory, features of the work environment shape employees’ attention to, interpretation of, and affective appraisal of events, thereby influencing the intensity and persistence of emotional reactions (Bissing-Olson et al., 2013). Employees regulate their emotional responses based on their appraisal of the meaning and significance of work events (Weiss and Cropanzano, 1996). Prior research further indicates that different organizational climates can lead to substantial variation in employees’ affective and behavioral responses to the same leadership behaviors (Kim et al., 2023; Kim et al., 2024). Existing empirical evidence suggests that strong situational cues may attenuate leaders’ influence on employees’ affective reactions, particularly in highly institutionalized environments where stable and consistent norms can weaken or even partially substitute for leadership-driven emotional processes (Dumont et al., 2017; Pawar and Eastman, 1997; Velez and Neves, 2018; Weiss and Cropanzano, 1996). As a shared perception of organizational support for environmental sustainability reflected in formal policies, practices, and informal norms (Norton et al., 2014), PEOC aligns closely with the organizational context factors described in Affective Events Theory that regulate affective responses. Moreover, this perspective is consistent with the broader leadership literature, which suggests that the effectiveness of leadership behaviors depends heavily on the organizational climate and situational features in which they are embedded. Within the framework of Affective Events Theory, different levels of PEOC are therefore expected to differentially moderate the indirect effect of GTL on EGB through EEP. Although GTL is generally regarded as a positive drive of EEP, in organizational contexts characterized by high PEOC, organizations already convey strong pro-environmental signals through explicit rules, stable value advocacy, and consistent behavioral expectations. These signals substantially elevate the baseline level of employees’ green behavior, such that employees’ environmental passion is already relatively high. Consequently, employees are less reliant on leadership-triggered affective events as a primary source of action motivation. Under these conditions, leaders’ green transformational initiatives may be perceived as excessive intervention in employees’ autonomous environmental engagement, making employees’ environmental passion more difficult to further activate. As a result, the marginal effectiveness of translating environmental passion into actual green behavior is reduced, weakening the affective pathway through which leadership promotes employees’ green behavior and leading to diminishing marginal returns. In contrast, in organizations with low levels of PEOC, where external institutional guidance and peer cues are relatively weak, employees are more dependent on affective events triggered by leaders to establish and sustain emotional commitment to environmental goals. In such contexts, employees’ environmental passion tends to be relatively insufficient, and the effect of GTL in enhancing EEP becomes more pronounced. The activated environmental passion not only strengthens employees’ internal identification with green goals but also enhances their motivation to convert emotional investment into concrete green behaviors, thereby more effectively encouraging proactive and persistent green behavior in daily work activities. Building on this reasoning, we advance the following hypothesis.

Hypothesis 3 (H3): PEOC negatively moderates the indirect effect of GTL on EGB via EEP, such that when PEOC is high, the marginal indirect effect of GTL on EGB through EEP is diminished.

### 2.4 Theoretical model

**Figure 1:**
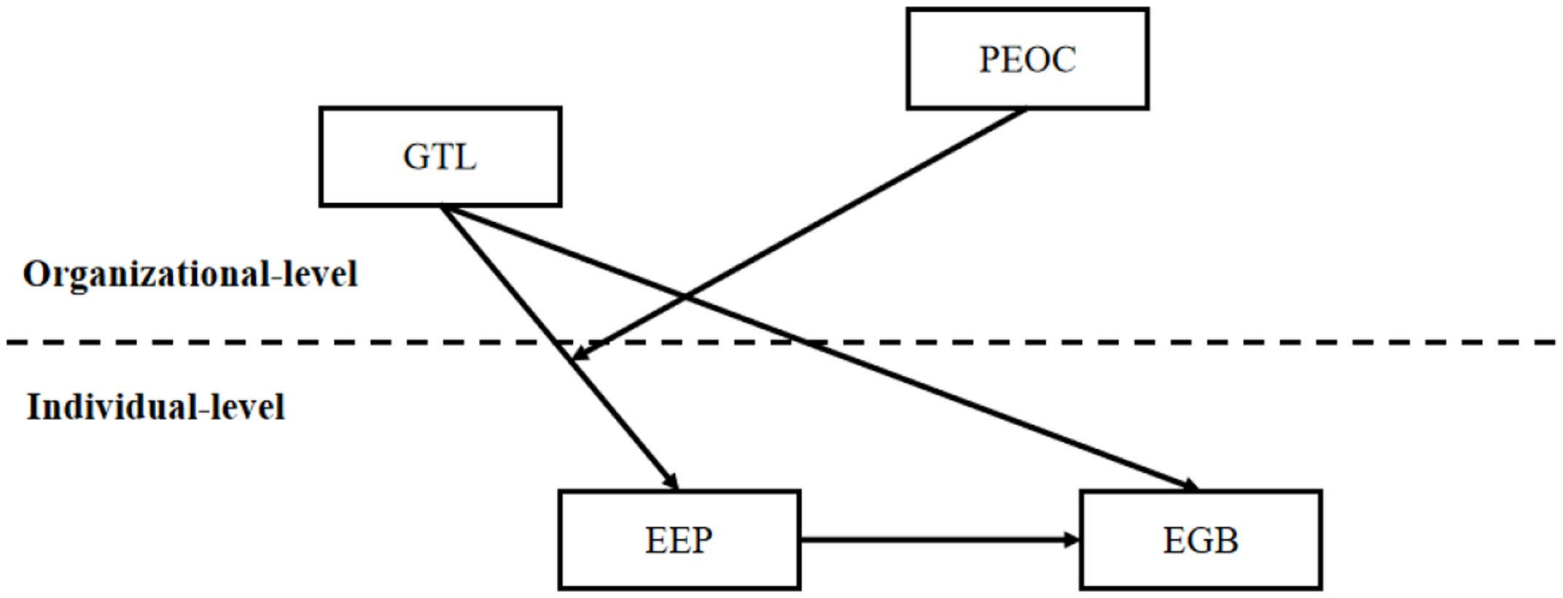
Theoretical model.

Based on the hypotheses developed above, this study constructs the theoretical model, which is illustrated in Figure 1.

## 3 Research design

### 3.1 Data collection

The survey was administered electronically and targeted employees from Luoyang Chanfu Food Co., Ltd., Luoyang Hengshu Trading Co., Ltd., Guoxin Futures Co., Ltd. (Luoyang Branch), Luoyang Jialijie Plastic Products Factory, and Henan Jiashu Health Technology Co., Ltd. Because GTL and PEOC are organizational-level constructs, survey items were differentiated between leaders and staff and responses were matched at the team level. Teams were organized based on work tasks and comprised between 3 and 10 members. Prior to data collection, participants were informed of the academic purpose of the study and assured that their participation was entirely voluntary. Informed consent was obtained from all participants before they proceeded to complete the questionnaire. The study involved minimal risk and did not collect any sensitive personal information. According to relevant institutional and national guidelines, ethical approval was not required for this type of anonymous, non-interventional survey research. Nonetheless, the study strictly adhered to established ethical principles for research involving human participants. All questionnaires were completed anonymously to ensure respondents’ candid answers. No identifying information (e.g., names, employee IDs, or contact details) was collected. The data were used exclusively for academic research purposes, stored securely, and accessed only by the research team. Confidentiality was maintained throughout the data collection, analysis, and reporting processes. The initial organizational sample consisted of 62 teams, from which a total of 380 questionnaires were collected. After excluding invalid questionnaires based on both item-level and overall validity checks, the final sample included 53 valid team units (valid response rate = 85.4%) and 325 valid employee questionnaires (valid response rate = 85.5%). The average team size was 6.1 members.

### 3.2 Measurement instruments

Given that employees have the most direct and accurate perceptions of their work context and personal affective experiences, both leadership-level variables (GTL and PEOC) and individual-level variables (EEP and EGB) were assessed based on employees’ self-reports. All constructs were measured using established scales originally developed by Western scholars. To ensure the scales’ applicability in the Chinese context, we employed a translation–back-translation procedure to produce Chinese versions of the instruments (Brislin, 1986). All items were rated on a five-point Likert scale, with higher scores indicating greater agreement with each statement.

#### 3.2.1 Employee green behavior

EGB was measured with the unidimensional scale developed by Robertson and Barling (2013). The scale comprises 7 items. A representative item is: “I suggest managers should adopt environmentally friendly practices to improve the organization’s environmental performance.”

#### 3.2.2 Green transformational leadership

GTL was measured using the multidimensional scale developed by Robertson and Barling (2013). The scale includes four dimensions—environmental influence, inspirational environmental motivation, environmental intellectual stimulation, and environmental individualized consideration—with a total of 12 items. A representative item is: “My leader serves as an environmental role model.”

#### 3.2.3 Employees’ environmental passion

EEP was measured using the unidimensional scale developed by Robertson and Barling (2013), which contains 10 items. A representative item is: “I enjoy engaging in environmentally friendly behaviors.”

#### 3.2.4 Pro-environmental organizational climate

PEOC was measured with the two-dimensional scale developed by Norton et al. (2014), which covers the organizational and coworker dimensions and comprises 8 items. A representative item is: “My company is committed to further environmentally friendly development.”

#### 3.2.5 Control variables

Prior studies indicate that demographic variables—such as gender, age, education level, position, tenure, company ownership type, industry affiliation, company size, and engagement in green-related research—are associated with EGB (Farrukh et al., 2022; Hameed et al., 2021; Huang et al., 2023). Accordingly, these demographic variables were included as control variables in the analyzes.

## 4 Data analysis and hypotheses test

### 4.1 Sample characteristics

The survey yielded 325 valid individual responses from 53 valid teams. The sample composition is summarized as follows. Gender: 56% male and 44% female, indicating no substantial gender imbalance. Age distribution: the sample skews relatively young, with 68% of respondents aged under 35. Education: the vast majority of respondents (95.7%) hold a bachelor’s degree or below. Organizational position: frontline employees account for the largest share (47.7%), whereas senior managers represent only 9.5%. Organizational tenure: 76.6% of respondents have been with their current employer for less than 5 years, whereas only 11.1% have ten or more years of tenure. Relation to the green industry: 41.8% of respondents’ majors are unrelated to environmental protection, and 38.7% work in industries that are not subject to national “total pollutant emission control” policies, indicating that most respondents’ professional backgrounds and industries have limited direct connections to environmental protection. Industry sector: the sample is concentrated in the service sector (58.5%), followed by high-technology sectors (18.8%). Ownership: respondents are primarily employed in state-owned and privately owned enterprises, which together account for 75.2% of the sample. Company Size: proportions are relatively balanced across size categories but show a tendency toward smaller enterprises. Overall, the sample distribution is reasonably balanced and exhibits satisfactory representativeness for the study’s purposes.

### 4.2 Variance decomposition and aggregation justification

Given the multilevel structure of the study (employee level nested within organization/team level), cross-level analytical procedures were adopted. We first estimated an empty (null) model to examine variance partitioning and to determine whether multilevel modeling was warranted. The null model yielded an intraclass correlation coefficient ICC(1) = 0.585, with within-group variance of 0.111 and between-group variance of 0.156. These results indicate that 58.5% of the variance in EGB is attributable to between-group (organizational) differences, thereby justifying the use of multilevel linear modeling. Because the organizational-level constructs—GTL and PEOC—were measured at the individual level, we evaluated the appropriateness of aggregating these measures to the organizational/team level. Common aggregation diagnostics include: ICC(1) > 0.05, ICC(2) > 0.50, and mean RWG > 0.70. The computed indices are as follows: for GTL, ICC(1) = 0.66, ICC(2) = 0.91, and mean RWG = 0.97; for PEOC, ICC(1) = 0.58, ICC(2) = 0.87, and mean RWG = 0.98. All indices exceed conventional thresholds, supporting the aggregation of individual responses to organization/team-level constructs.

### 4.3 Reliability and validity analysis

#### 4.3.1 Reliability analysis

Cronbach’s Alpha coefficients for all scales are reported in Table 1. Each alpha exceeds 0.70, indicating acceptable internal consistency reliability for all measurement scales.

**Table 1:** Reliability analysis.

Variable	Items	Cronbach’s Alpha
GTL	12	0.905
EGB	7	0.92
EEP	10	0.891
PEOC	8	0.923

#### 4.3.2 Validity analysis

(1) Common method bias test.

We assessed common method variance using Harman’s single-factor test. The first principal component accounted for 42.49% of the total variance, which is below the commonly used threshold of 50%. This result suggests that common method bias is unlikely to be a serious concern in the present dataset.

(2) Confirmatory factor analysis.

We conducted confirmatory factor analysis (CFA) to assess the discriminant validity of the measurement model by comparing the fit of the hypothesized multi-factor model with several alternative models in which different factors were combined. The fit indices for all competing models are reported in Table 2. The hypothesized four-factor model yielded the best fit: *χ*^2^ = 932.414, df = 428, *χ*^2^/df = 2.179, *p* < 0.001, Comparative Fit Index (CFI) = 0.902, Normed Fit Index (NFI) = 0.834, and Root Mean Square Error of Approximation (RMSEA) = 0.06. By contrast, the single-factor model showed the poorest fit: *χ*^2^ = 1592.449, df = 629, *χ*^2^/df = 2.532, *p* < 0.001, CFI = 0.845, NFI = 0.769, RMSEA = 0.069. Taken together, the CFA results indicate that the proposed four-factor measurement model demonstrates superior discriminant validity relative to alternative specifications, and the measurement structure is unlikely to be driven by a single common factor.

**Table 2:** Confirmatory factor analysis.

Model	*χ* ^2^	df	*χ*^2^/df	CFI	NFI	RMSEA
Four-factor model (A, B, C, D)	932.414	428	2.179	0.902	0.834	0.06
Three-factor model 1 (A + B, C, D)	1482.931	626	2.369	0.862	0.785	0.065
Three-factor model 2 (A, B + C, D)	1477.086	626	2.36	0.863	0.786	0.065
Three-factor model 3 (A, B, C + D)	1393.094	626	2.225	0.877	0.798	0.061
Two-factor model 1 (A + B + C, D)	1541.815	628	2.455	0.853	0.776	0.067
Two-factor model 2 (A, B + C + D)	1523.668	628	2.426	0.856	0.779	0.066
One-factor model (A + B + C + D)	1592.449	629	2.532	0.845	0.769	0.069

A = GTL; B=PEOC; C = EEP; D = EGB.

#### 4.3.3 Descriptive statistics and correlation analysis

Table 3 presents the descriptive statistics and Pearson correlation coefficients for the focal variables. Descriptive results indicate generally high levels of pro-environmental perceptions and behaviors in the sample. Specifically, GTL: mean = 4.32, SD = 0.47, indicating that respondents on average perceive their leaders’ green transformational behaviors as relatively strong. EGB: mean = 4.43, SD = 0.49, suggesting a high and relatively consistent level of self-reported green behavior. EEP: mean = 4.38, SD = 0.47, reflecting substantial affective engagement with environmental issues. PEOC: mean = 4.34, SD = 0.49, indicating that employees generally perceive strong organizational norms and support for environmental sustainability. Pearson correlation analysis (two-tailed) was used to examine relationships among variables, with significance reported using *p*-values. As shown in Table 3, the core variables are positively and significantly correlated. EGB and GTL: *β* = 0.754, *p* < 0.01; EEP and GTL: *β* = 0.829, *p* < 0.01; EEP and EGB: *β* = 0.819, *p* < 0.01; PEOC and GTL: *β* = 0.763, *p* < 0.01; PEOC and EGB: *β* = 0.681, *p* < 0.01; PEOC and EEP: *β* = 0.788, *p* < 0.01. In addition, to further address potential multicollinearity concerns arising from the high correlations among variables, we examined variance inflation factors (VIF) and condition index (CI) (Belsley et al., 1980). The VIF values for all variables were below 10, and all CI were also below 10, falling within acceptable thresholds. These results indicate that multicollinearity is not a serious concern in the present study. Overall, these results provide preliminary empirical support for the hypothesized relationships among GTL, EEP, PEOC, and EGB.

**Table 3:** Descriptive statistics and correlation analysis.

Variables	Mean	SD	1	2	3	4	5	6	7	8	9	10	11	12	13	14
Gender	1.44	0.50	1													
Age	2.09	0.95	0.122*	1												
Education	2.25	0.87	0.084	−0.059	1											
Position	3.13	1.00	0.065	−0.115*	−0.054	1										
Organizational tenure	1.85	1.02	−0.036	0.499**	−0.092	−0.080	1									
Major	1.60	0.49	−0.028	0.054	−0.096	0.306**	0.075	1								
Relation to green industry	1.42	0.49	0.077	0.172**	−0.147**	0.252**	0.220**	0.178**	1							
Industry sector	3.18	0.87	0.074	0.045	0.027	0.009	−0.056	0.151**	0.027	1						
Ownership	2.34	1.09	−0.025	−0.049	−0.095	0.031	−0.095	−0.008	−0.006	0.086	1					
Company Size	2.00	1.04	0.107	0.124*	0.092	0.018	0.214**	−0.042	0.282**	0.085	0.033	1				
GTL	4.32	0.47	−0.068	−0.030	0.118*	−0.116*	−0.052	−0.078	−0.139*	−0.002	−0.189**	−0.262**	1			
EGB	4.43	0.49	0.008	0.002	0.100	−0.090	0.030	−0.017	−0.044	−0.047	−0.274**	−0.105	0.754**	1		
EEP	4.38	0.47	−0.007	−0.012	0.082	−0.129*	−0.044	−0.055	−0.093	−0.003	−0.229**	−0.174**	0.829**	0.819**	1	
PEOC	4.34	0.49	−0.032	0.097	0.045	−0.188**	0.013	−0.120*	−0.095	0.054	−0.209**	−0.085	0.763**	0.681**	0.788**	1

**Indicates significance at the 0.01 level; *indicates significance at the 0.05 level.

### 4.4 Hypothesis testing results

#### 4.4.1 Test of the Main effect

Model 1 in Table 4 shows a significant positive effect of GTL on EGB (*β* = 0.993, *p* < 0.01). Therefore, H1 is supported, indicating that GTL promotes the occurrence of EGB.

**Table 4:** Analysis resulttables of main, mediation, and moderation effects.

Category	EGB				EEP	
	Model 1	Model 2	Model 3	Model 4	Model 5	Model 6
Individual-level						
Education	0.066	0.001	0.061	−0.146	−0.012	0.030
Position	0.199	0.171	0.225*	0.266	0.084	0.033
Organizational tenure	0.096	0.071	0.096	0.720	0.045	0.001
Major	0.092	−0.004	−0.253	−4.792	0.171	0.096
EEP		0.677**		0.667**		
EGB						
Organizational-level						
Ownership	−0.044*	−0.026	−0.046*	−0.137**	−0.026	−0.027
GTL	0.933**	0.274**	0.528**	0.109	0.962**	0.629**
POEC			0.300	0.260		0.253**
GTL × PEOC			−0.097**			−0.078**
EEP × PEOC				−0.032		
*R* ^2^	0.547	0.703	0.553	0.711	0.622	0.636
DW	1.90	2.01	1.92	2.16	1.97	2.04
*F*	513.003**	990.031**	435.930**	743.867**	836.068**	685.583**

**Indicates significance at the 0.01 level; *indicates significance at the 0.05 level.

#### 4.4.2 Test of the mediation effect

Model 5 in Table 4 indicates a significant positive relationship between GTL and EEP (*β* = 0.966, *p* < 0 0.01). When EEP was included in Model 2, EEP had a significant positive effect on EGB (*β* = 0.677, *p* < 0 0.01), and GTL remained significantly and positively related to EGB (*β* = 0.274, *p* < 0 0.01). The direct effect remained significant, suggesting partial mediation. Further, we estimated confidence intervals using a Bayesian two-level model with Markov chain Monte Carlo (MCMC) estimation. As shown in Table 5, the total indirect effect was 0.650 and was significant at the 1% level; the 95% confidence intervals was [0.535, 0.776], which does not include zero. After controlling for EEP, the direct effect of GTL on EGB was 0.274 and was significant at the 1% level; the 95% confidence intervals was [0.132, 0.418], which also does not include zero. Thus, H2 is supported, indicating that EEP partially mediates the relationship between GTL and EGB.

**Table 5:** Mediation effect confidence intervals.

Path	Effect size	SE	95% CI	
			Lower	Upper
GTL → EEP (a)	0.962	0.051	0.862	1.063
EEP → EGB (b)	0.677	0.053	0.573	0.782
Total indirect effect (a*b)	0.650	0.062	0.535	0.776
GTL → EGB (direct)	0.274	0.073	0.132	0.418

#### 4.4.3 Test of the moderated mediation effect

**Figure 2:**
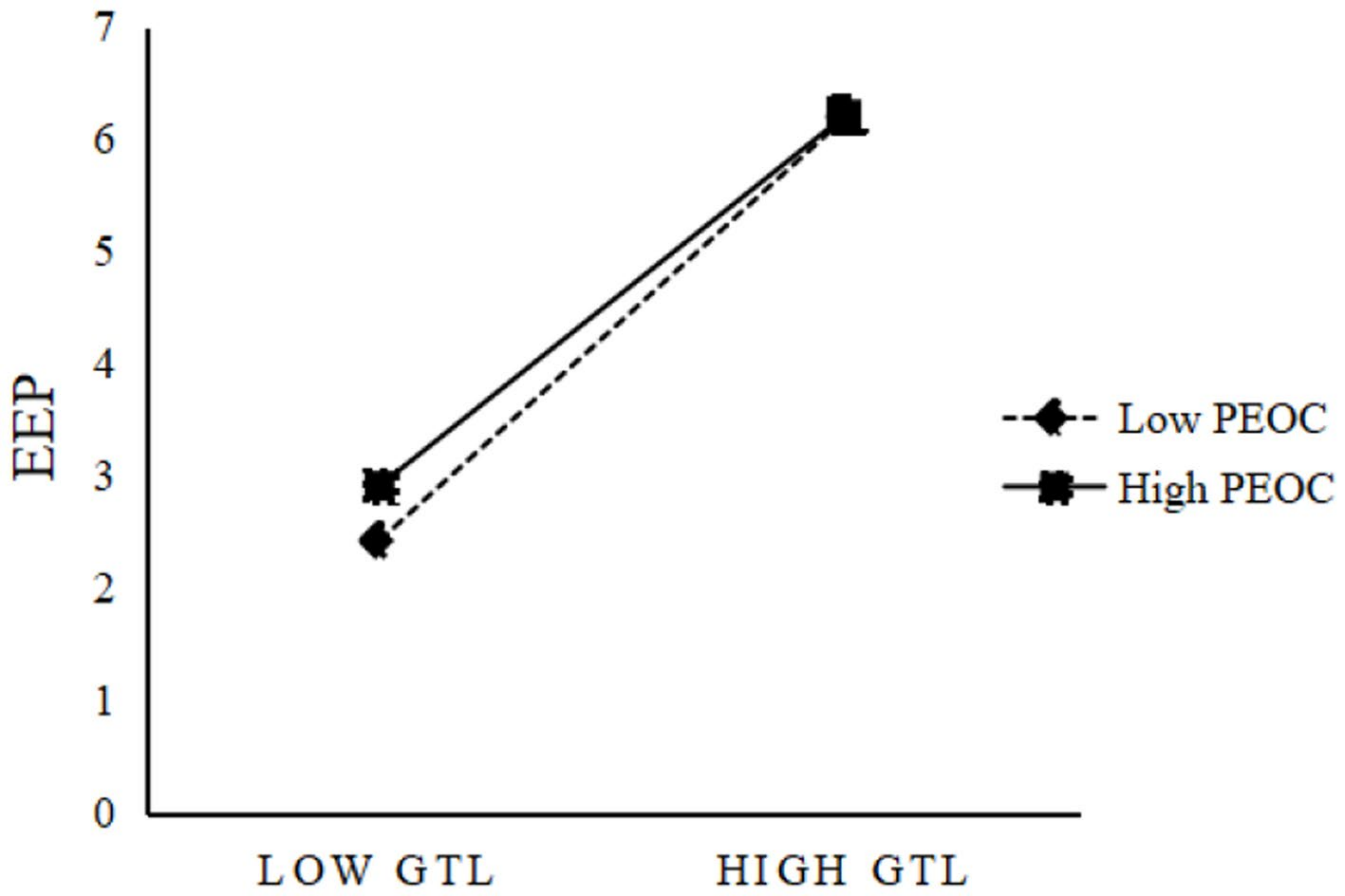
Simple slope analysis plot of the moderation effect of PEOC.

Building on Edwards and Lambert (2007), we tested a moderated mediation model using Mplus. Prior to constructing the interaction terms, the independent and moderating variables were grand-mean centered to reduce potential multicollinearity and facilitate interpretation. Given the theoretical assumption of homogeneous effects across groups and considerations related to sample size and model stability, the multilevel analyzes specified random intercepts with fixed slopes, and random slopes were not included. As shown in Table 4, Model 3 indicates that GTL positively predicts EGB (*β* = 0.528, *p* < 0.01), while the GTL × PEOC interaction negatively predicts EGB (*β* = −0.097, *p* < 0.01), satisfying the conditions for probing moderated mediation. Consistent with the proposed indirect pathway, Model 4 indicates that EEP positively predicts EGB (*β* = 0.667, *p* < 0.01), and Model 6 reveals a negative effect of the GTL × PEOC interaction on EEP (*β* = −0.078, *p* < 0.01). Thus, one set of coefficients necessary for moderated mediation is significant: the GTL × PEOC interaction negatively affects EEP, and EEP positively affects EGB. Simple-slope analyzes, conducted at PEOC values one standard deviation below and above the mean, further illustrate the moderating pattern (Figure 2): as PEOC increases, the positive effect of GTL on EEP weakens, providing support for H3. To quantify the conditional indirect effects, we employed MCMC-based confidence intervals; as shown in Table 6, the indirect effect decreases from 0.396 at low PEOC to 0.356 at high PEOC, with a difference of −0.040 and the 95% confidence intervals was [−0.074, −0.007], excluding zero. Taken together, these results support H3 and indicate that the mediating effect of EEP is negatively moderated by PEOC—that is, the indirect effect of GTL on EGB via EEP is weaker at higher levels of PEOC.

**Table 6:** Confidence intervals for the moderated mediation model.

Group/condition	Effect size	SD	95CI	
			Lower	Upper
Low PEOC	0.396	0.077	0.254	0.554
Medium PEOC	0.376	0.077	0.232	0.534
High PEOC	0.356	0.079	0.209	0.517
Difference	−0.040	0.017	−0.074	−0.007

## 5 Conclusions and discussion

### 5.1 Research conclusions

With the goal of advancing corporate sustainability, this study drew on social learning theory and affective events theory to develop and test a moderated mediation model linking GTL to EGB. Using multilevel regression analyzes based on data from 53 teams and 325 subordinates, we identified three principal findings. First, GTL exerted a significant positive effect on EGB (H1), this finding is consistent with Social Learning Theory, suggesting that employees develop EGB by observing, imitating, and internalizing leaders’ values, attitudes, and behavioral demonstrations. Second, EEP played a partial mediating role in transmitting the effect of GTL to EGB (H2), this result supports Affective Events Theory, indicating that leadership behaviors can function as salient affective events that elicit and sustain employees’ sustained EEP, which in turn facilitates the enactment of EGB. Third, the PEOC at the organizational level negatively moderated this mediating pathway, such that higher levels of PEOC weakened the indirect effect of GTL on EGB via EEP (H3), this finding extends Affective Events Theory by revealing the complexity of the GTL–EGB relationship under specific cultural conditions. A plausible explanation is that: in China, under a culture of high collectivism, employees are more inclined to comply with organizational norms and are more susceptible to PEOC, thereby weakening the marginal effect of GTL on EEP, resulting in a reduced mediating effect of EEP on GTL and EGB.

### 5.2 Managerial implications

To cultivate EGB and advance organizational green sustainability, firms should leverage GTL, stimulate EEP, and institutionalize coordination through a PEOC, thereby establishing systematic mechanisms for fostering EGB. Accordingly, several practical managerial implications can be derived. First, organizations should prioritize the development and effective utilization of GTL. To advance sustainability goals, organizations should implement management practices such as recruiting leaders with green transformational orientations, evaluating and rewarding leaders’ green performance, and enhancing leadership capabilities through green training and development programs. Second, organizations should actively stimulate EEP. GTL can foster affective events to energize EEP, such as leading by example and reinforcing shared environmental goals, which in turn promotes spontaneous green behavior. In the short term, leaders can rapidly activate EEP through vision articulation and emotionally engaging communication. In the medium term, organizations should provide resources and situational incentives to translate environmental passion into concrete behavior. In the long term, leadership behaviors and employees’ green practices should be embedded into formalized organizational systems to create a virtuous cycle. Third, organizations should attend to differences in their pro-environmental organizational climate. When seeking to enhance EGB through GTL, firms must carefully consider the existing level of PEOC. In organizations with relatively low PEOC, external institutional guidance and normative cues are insufficient to effectively direct employees’ behavior. Under such conditions, leadership interventions that stimulate environmental passion tend to yield greater marginal returns. Therefore, when PEOC is low, organizations can foster greater spontaneity in EGB and support sustainable development, by amplifying the indirect effect of GTL on EGB via EEP.

### 5.3 Limitations and future directions

While this study provides a new theoretical lens and empirical evidence on the effects of GTL on EGB, including its cross-level effects, it also has several limitations, and these findings should be interpreted with caution, which in turn suggest directions for future research. First, the sample was confined to China, which may not adequately represent employee behavioral patterns in other countries or regions. This constraint limits the generalizability of the findings, future research should consider cross-cultural comparisons to assess the applicability of the proposed model across different cultural contexts. Second, the theoretical model warrants further extension. While this study incorporates EEP, and PEOC, other potential mediating mechanisms, such as moral elevation (Rullo et al., 2022), green identity (Panda, 2023), and psychological safety (Ahmad and Umrani, 2019) or moderating variables, such as organizational structure (Fischer et al., 2019) and external institutional pressure (Jahanshahi and Al-Gamrh, 2024) may also play important roles in shaping EGB. Future research is encouraged to explore these alternative pathways and boundary conditions to develop a more comprehensive understanding of how GTL influences EGB.

## Data Availability

The original contributions presented in the study are included in the article/Supplementary material, further inquiries can be directed to the corresponding author.
